# The Diversity of Ribonuclease P: Protein and RNA Catalysts with Analogous Biological Functions

**DOI:** 10.3390/biom6020027

**Published:** 2016-05-13

**Authors:** Bradley P. Klemm, Nancy Wu, Yu Chen, Xin Liu, Kipchumba J. Kaitany, Michael J. Howard, Carol A. Fierke

**Affiliations:** 1 Department of Biological Chemistry, University of Michigan, Ann Arbor, MI 48109, USA; klemmbp@umich.edu (B.P.K.); kaitanyk@umich.edu (K.J.K.); mhowa@umich.edu (M.J.H.); 2 Program in Chemical Biology, University of Michigan, Ann Arbor, MI 48109, USA; nancywu@umich.edu; 3 Department of Chemistry, University of Michigan, Ann Arbor, MI 48103, USA; yyuchen@umich.edu (Y.C.); chemxliu@umich.edu (X.L.)

**Keywords:** RNase P, ribozyme, PRORP, endonuclease, tRNA maturation, tRNA recognition

## Abstract

Ribonuclease P (RNase P) is an essential endonuclease responsible for catalyzing 5’ end maturation in precursor transfer RNAs. Since its discovery in the 1970s, RNase P enzymes have been identified and studied throughout the three domains of life. Interestingly, RNase P is either RNA-based, with a catalytic RNA subunit, or a protein-only (PRORP) enzyme with differential evolutionary distribution. The available structural data, including the active site data, provides insight into catalysis and substrate recognition. The hydrolytic and kinetic mechanisms of the two forms of RNase P enzymes are similar, yet features unique to the RNA-based and PRORP enzymes are consistent with different evolutionary origins. The various RNase P enzymes, in addition to their primary role in tRNA 5’ maturation, catalyze cleavage of a variety of alternative substrates, indicating a diversification of RNase P function *in vivo*. The review concludes with a discussion of recent advances and interesting research directions in the field.

## 1. Introduction

**Figure 1:**
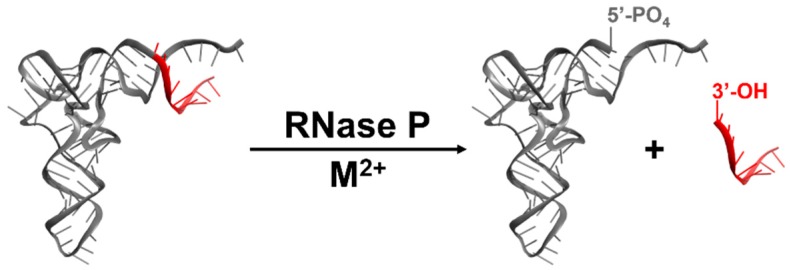
RNase P enzymes catalyze metal-dependent, endonucleolytic cleavage of pre-tRNA (adapted with permission from [7]).

The 1989 Nobel Prize in Chemistry was awarded to Sidney Altman and Thomas Cech “for their discovery of catalytic properties of RNA” [1]. Altman was recognized for determining that the ribonuclease P (RNase P) activity of *Escherichia coli* originates from an RNA subunit [2,3]. RNase P is a metal-dependent endonuclease that catalyzes phosphodiester bond hydrolysis of precursor transfer RNA (pre-tRNA), generating tRNA with a mature 5’ end, including a 5’ phosphate, and a 5’ leader with a 3’ hydroxyl (Figure 1) [4]. Pre-tRNAs are non-functional in translation and must be processed for protein synthesis to occur [5,6]. Therefore, RNase P plays a key role in cellular homeostasis and survival.

Two types of RNase P enzymes exist: (1) RNA-dependent enzymes (ribozymes), for which the active site is located in a catalytic RNA subunit, and (2) protein-only RNase Ps (PRORPs; also referred to as “proteinaceous RNase Ps” in various literature sources, we note that eukaryotic RNA-based RNase Ps are also proteinaceous, with ≥70% protein by mass, so “protein-only RNase P” is more precise and we favor this nomenclature). RNase Ps provide the opportunity to compare catalytic strategies of independently evolved protein and RNA catalysts in the only known biological model system in which both are utilized in extant biology to execute the same biological function.

In the following sections, we will review: the diversity and distribution of RNase Ps throughout life; the known structures of RNase P enzymes; the catalytic strategies employed by RNase P; and the substrate recognition strategies of RNase P. We conclude with a summary of unresolved questions and current directions within the field.

## 2. Diversity and Distribution of RNase Ps

**Figure 2:**
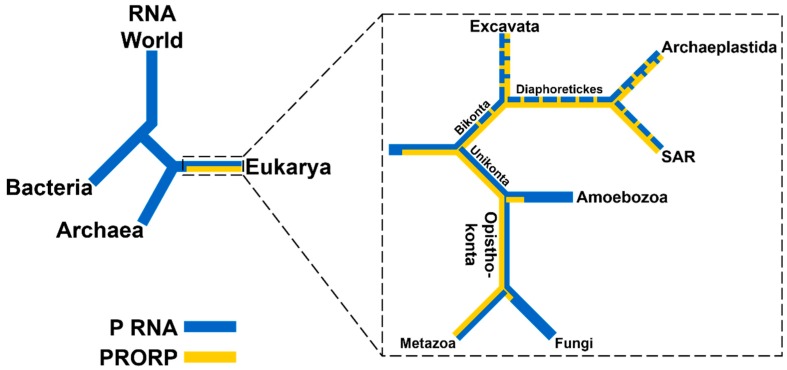
Distribution of RNase P enzymes across evolutionary lineages. Presence of a P RNA (blue) or PRORP (yellow) is indicated. **Left**: RNA-based RNase Ps are found in all domains of life and are proposed to have evolved from an RNA predecessor lacking protein components. PRORPs exist only in Eukarya and likely evolved after the divergence with Archaea. **Right**: Distribution of RNase Ps in Eukarya using the five supergroup model (aspects of the branching order remain controversial) [12,13]. PRORPs are found in four supergroups: Excavata, Archaeplastida, SAR [Stramenopiles, Aveolata, and Rhizaria], and Opisthokonta, but not in the fifth: Amoebozoa [11]. Dashed blue or yellow lines indicate that some clades within the supergroup lack RNase P RNA or PRORP sequences, respectively.

RNase P enzymes are found in all domains of life and in nearly all species. There is one known exception: the obligate symbiont *Nanoarchaeum equitans* does not encode pre-tRNAs with 5’ extensions and thus lacks an RNase P [8,9]. While RNA-based RNase Ps have been found in all three domains of life, PRORPs are found exclusively in eukaryotes (Figure 2) [10]. Given the broad distribution of PRORPs across Eukarya, in particular across lineages as diverse as trypanosomes, plants, and animals, it is likely that the proteins emerged in basal eukaryotes and prior to the divergence of supergroups. However, there are several eukaryotic lineages for which an RNase P enzyme has not been identified in the genome—*Jacobida* (Excavata), Cyanidiophyceae (Archaeplastida), and *Nuclearia* (Opisthokonta)—highlighted by an apparent lack of either PRORP or RNase P RNA sequences [11]. These species may have maintained a P RNA that is too distinct from the canonical sequence to be identified from genomic queries, or have genomic sequences that are currently too incomplete to allow identification of a P RNA or PRORP.

### 2.1. RNA-Based RNase Ps Exist in Bacteria, Archaea, and Many Eukarya

**Figure 3:**
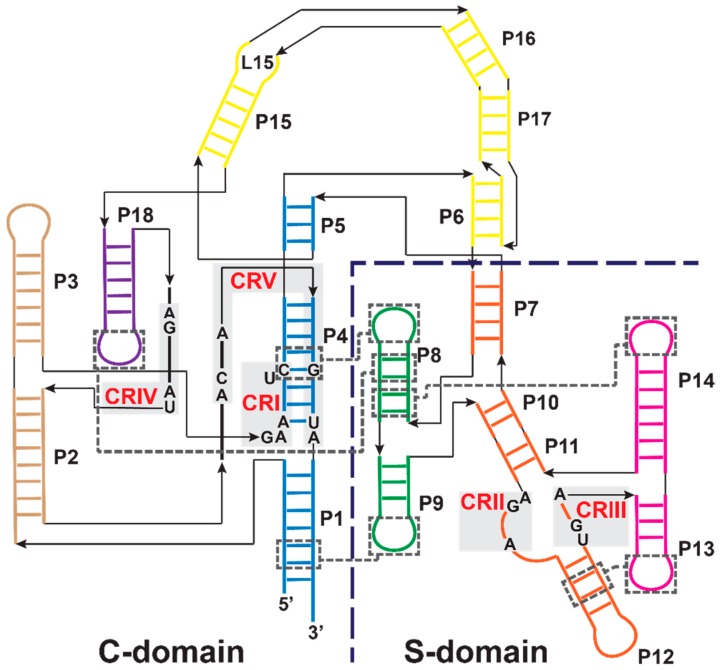
The secondary structure of type A RNase P RNA from *T*. *maritima* (adapted with permission from [21]) [22,23]. Individually-folding catalytic (C)- and specificity (S)-domains are divided by the dashed line. Conserved regions (CRI–V, red) are indicated by gray shading and numbered in order of occurrence from the 5’ end [4]. Tertiary interactions in the *T*. *maritima* P RNA are indicated by dashed gray boxes and lines. Helices are colored by coaxial-stack and are numbered as P1–P18 in order of occurrence from the 5’ end. The C-domain includes P1/P4/P5 (blue), P2/P3 (brown), P6/P15/P16/P17 (yellow,) and P18 (purple). The S-domain includes P7/P10/P11/P12 (orange), P8/P9 (green), and P13/P14 (pink).

RNA-based RNase Ps are ribonucleoprotein (RNP) complexes containing one conserved catalytic RNA subunit and a variable number of protein subunits depending on the organism. In general, protein content increases from ~ 10% to ~ 40% to ≥70% for the enzymes from Bacteria to Archaea to Eukarya, respectively. Unlike self-cleaving ribozymes, RNA-based RNase Ps catalyze multiple turnovers and remain unchanged after catalysis. Bacterial, archaeal, and eukaryotic nuclear RNase P RNAs share a core consensus sequence with a conserved secondary structure [14]. There are three distinct types of secondary structures in Bacteria: type A (ancestral), B (*Bacillus*), and C (Chloroflexi), and two in Archaea: M (Methanococci) and P (*Pyrobaculum*), while the eukaryotic nuclear RNase P RNA has a larger variety of secondary structures [15,16,17,18,19,20]. The secondary structure of a type A RNase P RNA from *Thermotoga maritima* is shown in Figure 3.

RNase P in Bacteria is composed of one large RNA subunit (P RNA), typically 340–420 nucleotides, and one small protein subunit (RnpA), approximately 14 kDa [24]. Both subunits of the bacterial ribozyme are essential for efficient catalytic activity *in vivo*, while the RNA subunit is sufficient for catalysis *in vitro* at high cation concentrations [3]. Phylogenetic analysis classified the structure of bacterial RNase P RNA into three types [16,25,26,27,28]. Type A is the most common bacterial P RNA, represented by the *Escherichia coli* RNase P RNA, while type B is found in most low-GC gram-positive Bacteria, with the *Bacillus subtilis* RNase P RNA as the archetypal example [16,29]. Type C RNAs are found in some Chloroflexi and appear to be intermediate between types A and B [16,17]. The secondary structures of types A and B are significantly different. However, the differences are not in the consensus regions, which contain the sequences and structures required for catalytic function and substrate selectivity *in vitro* [30].

The RnpA protein is approximately 10% of the bacterial holoenzyme by mass and enhances the affinity of RNase P for pre-tRNA and metal ions [31,32,33,34]. RnpA contacts the leader sequence of pre-tRNA to stabilize an active enzyme-substrate conformer [35]. The protein binds to the P RNA catalytic (C)-domain at P2, P3, junction (J)18/2, and conserved region (CR)V in J2/4 (Figure 3) [22,36]. The bacterial RnpA proteins share little sequence similarity, yet are structurally homologous to each other [37,38,39]. Bacterial RnpA proteins are functionally interchangeable; the RNA and protein subunits from one species can complement subunits from the other species with variable reactivity [3,40,41].

Archaeal RNase P RNAs most commonly resemble the ancestral bacterial type A, but lack the P13/P14 and P18 regions. *Methanococci* and *Archaeoglobus* encode type M RNAs, which are similar to archaeal type A RNAs but lack P8, P16, the P6 pseudoknot, and a substrate recognition loop in P15 [42]. Type P RNAs are found in *Pyrobaculum* and contain only the catalytic domain, lacking nearly the entire specificity domain [43]. Most archaeal RNase P RNAs are not catalytically active in the absence of proteins, with a few exceptions (such as P RNA from *Methanobacterium formicicum* and several *Methanobacterium thermoautotrophicum* strains) in 4 M ammonium acetate with 300 mM magnesium chloride [44].

Archaeal RNase P contains at least four proteins, with a fifth associating with type M RNA, all of which are homologous with yeast and human nuclear RNase P proteins [5,45,46,47,48]. The archaeal RNase P proteins make up at least 40% of the holoenzyme mass and share significant similarity to human nuclear RNase P proteins RPP21, RPP29, RPP30, RPP38, and POP5 [5,48,49]. In *Pyrococcus furiosus*, two binary subcomplexes form: RPP21–RPP29 and RPP30–POP5 (using the human nomenclature) [50]. Both complexes affect cleavage site selection, while RPP21–RPP29 also enhances substrate affinity [50]. In Type M RNAs, RPP38 binds to a kink-turn in P12, increasing the thermostability and the catalytic efficiency of this enzyme *in vitro* [47,51].

Eukaryotic RNA-based RNase Ps have been extensively studied in yeast and humans. In addition to homologs of the five archaeal proteins, the nuclear eukaryotic RNA-based RNase P includes at least four additional proteins in Fungi and at least five additional proteins in Metazoans, comprising >70% of the holoenzyme by mass [5]. *Saccharomyces* *cerevisiae* nuclear RNase P contains an RNA (RPR1) and nine protein subunits: POP1, 3, 4, 5, 6, 7, 8, RPP1, and RPR2, and has been purified and biochemically characterized *in vitro* [49]. All 10 subunits are essential for activity *in vivo* [49,52,53,54,55]. Fungi contain an additional mitochondrial RNA-based RNase P that consists of a single mitochondrial-encoded RNA subunit (RPM1), which has little similarity to the other RNase P RNAs and at least one large, unique nuclear-encoded protein component (RPM2), which has no sequence homology to other RNase P proteins [56,57].

Humans and other metazoans have two RNase P enzymes: a nuclear RNA-based RNase P and a mitochondrial PRORP. Human nuclear RNase P contains a single RNA subunit (H1 RNA) and at least ten protein subunits: RPP14, 20, 25, 29, 30, 38, 40, and hPOP1 and 5, seven of which are homologous to the protein subunits in yeast (Table 1) [5,58,59]. The human mitochondrial PRORP consists of three nuclear-encoded RNase P proteins (MRPP1, 2, and 3), none of which are homologous to the RNA-based RNase P proteins (see following section) [60]. H1 RNA has minimal catalytic activity in the absence of its associated protein subunits *in vitro* [61]; the apparent dissociation constant (*K*_D,app_) is increased 20-fold–50-fold and the single-turnover observed rate constant (*k*_obs_) is reduced by >10^5^ when compared to the bacterial RNase P RNA [61].

The alga *Ostreococcus tauri* encodes RNase P RNAs in its mitochondrial and plastid genomes, but not in the nuclear genome, which instead encodes a PRORP [62]. The RNase P RNAs associate with a nuclear-encoded bacterial-like P protein and catalyze pre-tRNA cleavage *in vitro* [62]. Further, the *O*. *tauri* RNA-based RNase P protein complements growth of *E*. *coli* that encode a bacterial P protein with a temperature-sensitive mutation at the restrictive temperature [62]. The retention of organelle-encoded P RNAs in Fungi and some algae indicate that these enzymes were present in basal eukaryotic lineages, but have been replaced by PRORP in clades as diverse as land plants and mammals.

### 2.2. PRORPs are Found Only in Eukarya

PRORP sequences are found broadly in the nuclear genomes of Eukarya, including animals (Metazoa), plants and algae (Archaeplastida), trypanosomes (Excavata), and heterokonts (SAR subgroup Stremenopiles) (Figure 2) (see [11] for a more complete discussion). PRORPs from the land plant *Arabidopsis thaliana* are able to complement viability in an *E*. *coli* strain with the RNase P RNA under control of the arabinose promoter when grown on glucose media, as well as a yeast strain lacking core protein components for the RNA-based RNase P, indicating that PRORPs can maintain the essential biological functions of the RNA-based RNase P [63,64]. Human mitochondrial RNase P (mtRNase P) was the first RNase P enzyme systematically shown to lack an RNA component [60]. This enzyme requires three MRPP subunits: tRNA methyltransferase 10 C (TRMT10C; MRPP1), a short-chain dehydrogenase/reductase (SDR5C1; MRPP2), and a novel metallonuclease, which houses the RNase P active site (huPRORP; MRPP3). All three subunits were affinity-purified and all were required to reconstitute efficient pre-tRNA cleavage activity *in vitro* [60]. SiRNA knockdowns of either MRPP1 or MRPP3 resulted in precursor transcript accumulation in mitochondria from HeLa cells [65].

MRPP3 represents a novel class of endonucleases, containing an N-terminal pentatricopeptide repeat (PPR) RNA-binding domain, a central structural Zn^2+^-binding domain, and a Nedd4-BP1, YacP nuclease (NYN) domain with homology to the flap endonucleases [66]. The molecular basis for the requirement of MRPP1 and MRPP2 for metazoan mtRNase P activity remains to be clarified. MRPP1 is one of three human homologs of Trm10, a SAM-dependent methyltransferase that catalyzes the addition of a methyl group to the *N*1 of G9 in yeast tRNAs [67]. MRPP1 and MRPP2 form a stable methyltransferase complex (MRPP1/2) which catalyzes the addition of a methyl group to the *N*1 of a purine (A or G) at position 9 in mitochondrial tRNAs [68]. Both pre-tRNA and mature tRNA are substrates for the MRPP1/2 complex [68]. However, this methyltransferase activity is not required for RNase P catalysis. The MRPP1/MRPP2 complex is proposed to enhance the affinity of MRPP3 for the substrate by protein–protein contacts, by reorganizing the tRNA structure for recognition by MRPP3 and/or for altering the structure of the MRPP3 active site [60,68,69].

Plants, algae, and trypanosomes encode homologs of MRPP3 that do not require additional subunits for catalysis *in vitro*. The algae, *O*. *tauri*, encodes one PRORP homolog that catalyzes pre-tRNA processing *in vitro* [62], trypanosomes encode two functional PRORP paralogs [70], and land plants encode three functional PRORP paralogs [63].

In *A*. *thaliana* (*At*), PRORP1 is localized to the mitochondria and chloroplasts, while PRORP2 and PRORP3 co-localize to the nucleus [63]. *At*PRORP2 and *At*PRORP3 have overlapping substrate selectivity, since depletion of both enzymes is required to eliminate RNase P activity in the nucleus [71]. They are highly similar, sharing 80% sequence identity and 88% similarity, suggesting a relatively recent gene duplication event. Given that it is not possible to obtain viable cells containing a deletion of PRORP1 or a double deletion of PRORP2/3, it was proposed that *A*. *thaliana* is devoid of an RNA-based RNase P [71]. We note that the *At*PRORP3 gene is currently misannotated in databases, with a translation start site at nucleotide 2 of the mRNA transcript. The translation start site was previously determined to be the second ATG, which results in a start site shifted to the second in-frame methionine, at the same position as *At*PRORP2 immediately prior to the nuclear localization sequence (NLS) [63].

In the simple moss *Physcomitrella patens*, the localization of three PRORPs was determined by GFP-fusions with N-terminal sequences [72]. Two PRORPs, designated *P*. *patens* pentatricopeptide repeat protein 67 (*Pp*PPR67) and *Pp*PPR104, co-localize to the organelles, while *Pp*PPR63 localizes to the nucleus [72]. Knockout of the nuclear-localized *Pp*PPR63 is not lethal, leading to the proposal that an alternative RNase P activity exists [72]. We note that *Pp*PPR67 contains a classical bipartite NLS (residues 222–249) downstream of the GFP-fusion sequence, which is nearly identical to the *Pp*PPR63 NLS (residues 1–28), suggesting the possibility of nuclear localization for both proteins [73]. The viability of *P*. *patens* with a dual knock-out of *Pp*PPR63 and *Pp*PPR67 was not reported [72]. Thus, it remains possible that *Pp*PPR67 has nuclear localization sufficient to compensate for the loss of *Pp*PPR63.

In some Excavata, such as the parasite *Trypanosoma brucei*, two differentially-localized PRORPs exist in place of an RNA-based RNase P. Initial detection of RNase P activity in *T*. *brucei* revealed no apparent RNA component and an apparent mass much less than typical RNA-based RNase Ps [74]. Subsequent characterization and purification of these enzymes revealed two PRORP homologs that catalyze RNase P cleavage *in vitro* and process pre-tRNAs in the nucleus (*Tb*PRORP1) and mitochondria (*Tb*PRORP2) [70].

## 3. Structures of RNase Ps

There have been a variety of structures solved for both RNA-based RNase Ps and PRORPs from all three domains of life including individual subunits, subcomplexes, and holocomplexes. In addition to visualizing features previously proposed from biochemical experiments, many structures have provided significant insight into structural features that are key for catalysis and substrate recognition.

### 3.1. Bacterial RNase P Ribozyme

The highly conserved core structure of bacterial RNase P RNAs includes two independently folding domains: the C- and S-domains (Figure 3). The C-domain comprises 60% of the P RNA and can catalyze pre-tRNA cleavage both in the presence and absence of the RnpA protein, although the cleavage rate constant and pre-tRNA substrate affinity are decreased compared to intact RNase P RNA [75,76,77]. The S-domain comprises the remaining 40% of the P RNA and is important for substrate affinity/specificity, interacting with the D- and TψC-domains of pre-tRNA [78,79,80]. The P RNA contains five regions of universal sequence conservation (CR) found throughout all RNase P RNAs, with CR-I, IV, and V in the C-domain and CR-II and III in the S-domain [14,81,82]. CR-I and V include nucleotides located within the P4 helix in the C-domain, which is the most conserved region of P RNA and possesses 11 of the 21 universally conserved nucleotides [4,14]. This region houses the active site and binds at least two catalytic metal ions (discussed in the next section).

**Figure 4:**
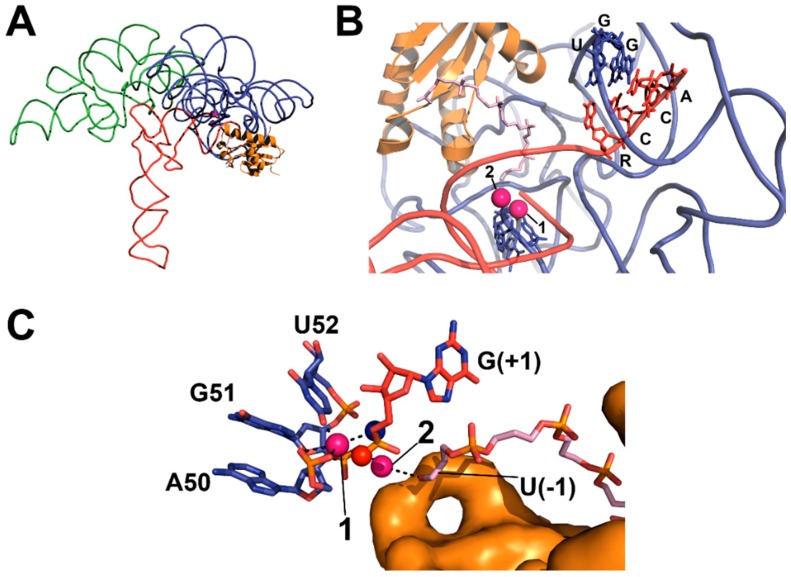
X-ray crystal structure of *T*. *maritima* RNase P-tRNA-leader product complex (PDB 3q1r, PyMOL) [22]. (**A**) The holoenzyme-product complex of RNase P is shown, including C- (blue backbone) and S- (green backbone) domains, tRNA (red backbone), and RnpA (orange cartoon); (**B**) Topology of substrate contact sites in the catalytic domain (colored as in A), including the 5’ leader (light pink sticks) bound to RnpA and proposed divalent metal ions (pink spheres). Base-pairing between GGU residues in P RNA and the 3’ RCCA of tRNA (U256–R(73), G255–C(74), G254–C(75)) is shown; (**C**) Topology of the active site (colored as in A), including the active site residues (blue carbon atoms), product G(+1) (red carbon atoms), the 5’ leader (light pink carbon atoms) bound to RnpA (orange surface), proposed metal contacts (black dashed lines), and positions of the pro-*R*_P_ (red sphere) and pro-*S*_P_ (blue sphere) oxygens of the product 5’ phosphate.

Structures of bacterial RNase P have revealed the spatial arrangement of the C- and S-domains, the RnpA-binding site, substrate interaction sites, and metal-binding sites. The X-ray crystal structures of RNA components from both type A (from *T*. *maritima*) and B (from *Bacillus stearothermophilus*) RNase Ps have been solved [83,84]. Structural analysis also revealed that the structure of RnpA does not change upon binding to P RNA [22,85,86,87]. RnpA has an overall αβββαβα topology with two RNA-binding regions: a central cleft and an RNR motif [85,86,87]. A crystal structure of the *T. maritima* RNase P holoenzyme, in a product complex with a yeast tRNA^Phe^ and a 5’ leader, validated the importance of the CR sequences (PDB ID: 3q1r, Figure 4A) [22]. CR-II and CR-III of the P RNA S-domain interact with the D- and TψC-loops of tRNA. CR-I, CR-IV, and CR-V regions in the C-domain include non-helical elements and contribute to interactions with the minor groove of the tRNA acceptor-stem.

Two metal-binding sites located in the active site near the P4 helix, previously identified through biochemical techniques, were validated by soaking the crystals with Eu^2+^ and Sm^3+^ (Figure 4B) [22]. However, Eu^2+^ and Sm^3+^ both vary in ionic radius and coordination geometry from the catalytic divalent metal ions, such as Mg^2+^, Mn^2+^, or Ca^2+^ [22,88]. Furthermore, the resolution of the crystal structure (4.2 Å) makes it difficult to identify ligand–metal contacts and distinguish between inner- and outer-sphere metal ion coordination. That said, metal 1 is located between the tRNA 5’ end, the phosphodiester bonds of A50 and G51 and the carbonyl oxygen O4 of U52, which is universally conserved. The metal 2 site is located close to the phosphoryl oxygens of G51, the 3’ OH of the leader, and the 5’ end of tRNA.

Other structural methods have been used to identify metal ion-binding sites in the active site of RNase P RNA. Anomalous scattering in diffraction analysis (3.5 Å resolution) of *B. stearothermophilus* RNase P RNA crystals soaked in Os(NH_3_)_6_^3+^ (which mimics Mg(H_2_O)_6_^2+^), Pb^2+^, Sm^3+^, Gd^3+^, or Yb^3+^ identified a number of possible metal ion-binding sites in RNase P with two metal ion-binding sites observed near residues A49, A50, G51, G318, and G319 (unless otherwise specified, bacterial RNase P RNA residues are numbered by the comparable *T*. *maritima* position for clarity) [89]. Additionally, nuclear magnetic resonance (NMR) data using a P4 helix stem-loop mimic suggested association of Mg^2+^ at residues corresponding to G318 and G319 in RNase P [90,91,92]. Metal-induced cleavage of a model of the P RNA L15 and phosphorothioate substitutions at those residues indicated that L15 binds to metal ions important for catalysis [93].

### 3.2. Archaeal and Eukaryotic RNA-Based RNase Ps

There are no atomic resolution structures of full-length RNase P RNA from Archaea or Eukarya. However, the overall fold is proposed to be similar to the RNA component of the bacterial enzyme based on nucleotide identity and secondary structure conservation, especially in regions pertaining to substrate recognition and enzymatic catalysis. Structures have been solved for each of the five archaeal RNase P protein subunits: RPP21 [94,95], RPP29 [96,97], RPP30 [98], RPP38 [99,100], and POP5 [101] (using the human nomenclature). X-ray crystallography and NMR spectroscopy have both revealed the three-dimensional structure of the RPP21–RPP29 subcomplex [102,103]. Footprinting experiments revealed that the RPP21–RPP29 subcomplex contacts the S domain, while the RPP30–POP5 subcomplex contacts the catalytic domain of archaeal RNase P RNA in a position similar to the bacterial protein [39,103].

Two yeast RNase P proteins, RPP20 and RPP25, were solved in complex with the P3 domain of the RNA subunit using X-ray crystallography to 2.7 Å [104]. Furthermore, electron microscopy revealed low resolution structural information about the *Saccharomyces cerevisiae* RNase P holoenzyme [105], providing additional information about the location of the protein subunits. This included validation of the RPP20–RPP25 interactions with P RNA helix P3, as well as modelling POP5–RPP30 near the C-domain as is the case in archaeal and bacterial RNase P [105].

### 3.3. Protein-Only RNase Ps

X-ray crystal structures have been solved for both the single-subunit plant PRORPs and the multi-subunit metazoan PRORPs. In the single-subunit plant enzymes, the structures of *A*. *thaliana* PRORP1 and PRORP2 visualized similar folds [66,106]. Two structures of huPRORP/MRPP3 were recently reported, both with extensive truncations in the PPR domains [69,107]. Structures of the MRPP2, which is a homotetramer in solution and *in crystallo*, have been solved [108]. No structures of MRPP1 have been solved, but there are several partial structures of homologous Trm10 family tRNA methyltransferases [109].

**Figure 5:**
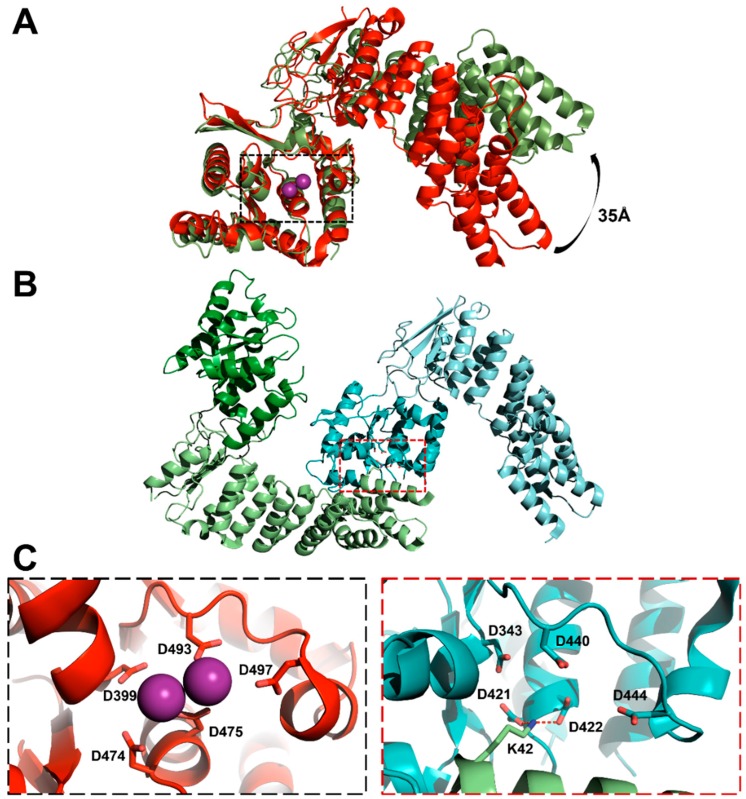
Crystal structures of *At*PRORP1 (PDB 4g24) and *At*PRORP2 (PDB 5diz) [64,103]. (**A**) Three-dimensional alignment of *At*PRORP1 and *At*PRORP2 structures via their active sites (dashed box). PRORP1 (red) topology includes the NYN metallonuclease domain bound to Mn^2+^ (purple spheres). PRORP2 (green) is in a more “open” conformation, resulting in a ~35 Å difference in the position of the first PPR helix; (**B**) *At*PRORP2 crystallization dimer (left), with subunits labeled in green and cyan (NYN domains in brighter colors); (**C**) Expanded views of the dashed boxes in panel A (left) or B (right). The *At*PRORP1 active site (left) includes four fully-conserved aspartates and one partially-conserved aspartate (sticks). Close-up of intersubunit interaction (right) between *At*PRORP2 molecules, with active site aspartates of one molecule interacting with Lys 42 (sticks) of the second (red dashed lines).

The crystal structure of *A*. *thaliana* PRORP1 revealed a three-domain architecture, with an NYN metallonuclease domain, a bipartite central Zn^2+^-binding domain, and a PPR RNA-binding domain (Figure 5A) [66]. The active site, located within the NYN domain, contains four aspartates (*At*PRORP1 Asp 399, 474, 475, and 493) that are fully conserved across the available PRORP sequences, with a fifth aspartate (*At*PRORP1 Asp 497) not conserved in some metazoan homologs. The structure of *At*PRORP1 bound to Mn^2+^ (PDB ID: 4g24), which activates the enzyme, revealed density for two metal ions bound in the active site [66]. Mechanistic studies with *At*PRORP1 have provided further evidence for a two-metal ion mechanism [110], which is discussed in the following section.

The crystal structure of *At*PRORP2 revealed a more “open” conformation, in which the activesite and PPR domain are rotated away from one another (Figure 5A) [106]. Interestingly, the PRORP2 monomers packed in an extended chain, with a conserved lysine (*At*PRORP2 Lys 42) from one chain inserted into the active site of the neighboring chain (PDB ID: 5diz). While no metal densities were obtained by soaking metals into the crystal, the construct was active and monomeric in solution [106]. Thus, the dimerization interactions *in crystallo* are not likely to be relevant to *in vivo* function.

**Figure 6:**
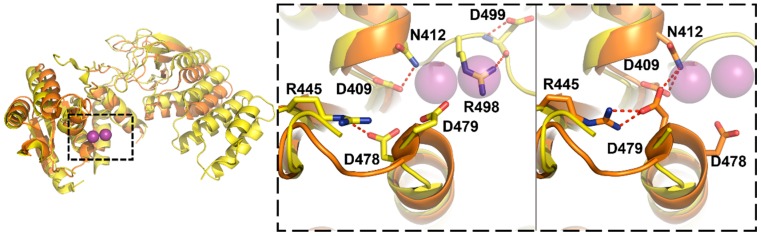
Alignment of two MRPP3 structures (PDB 4xgl, yellow; PDB 4rou, orange) with the *At*PRORP1 active site (PDB 4g24, only metal ions shown) [66,69,107]. **Left**: Alignment of two MRPP3 structures with the PRORP1 active site (purple spheres). MRPP3 NYN residues (4xgl: S361/P362, D409, D479, and D499, or 4rou: residues S361/P362, D409, D478, and D479) were aligned to the equivalent PRORP1 residues. The dashed box is expanded on the right. Middle and Right panels: Dashed box contains the aligned MRPP3 active site residues from 4xgl (middle, yellow) or 4rou (right, orange) with Mn^2+^ from PRORP1 (transparent purple spheres). Residues with the potential to occlude the metal sites, N412, R445, and R498, are shown if coordinates are available. Hydrogen bonds between residues are shown as dashed red lines.

Two recent crystal structures revealed a V-shaped structure for MRPP3 comparable to *At*PRORP1 (PDB ID: 4xgl, 4rou; Figure 6) [69,107]. The MRPP3 constructs used in both structures include truncation of two or four of the PPR motifs. Both structures contain significant disorder in the NYN domain, the metal 1 site is occluded by asparagine 412, and arginine 445 distorts the positions of D478-D479 (Figure 6, middle/right) [69,107]. The structure reported by Reinhard, *et al*. [69] also visualizes R498, which forms a hydrogen bond with D499—the equivalent of the metal ligand D399 in *At*PRORP1–and occludes the metal 2 site (Figure 6, middle) [69].

The authors independently ascribe the NYN domain disorder and active site occlusion as an inactive conformation that prevents metal binding, and suggest that the MRPP1/2 complex activates catalysis by promoting an active conformation [69,107]. However, even the more complete MRPP3 construct was inactive in assays containing MRPP1/2 [69], so the distorted active sites might also be related to the N-terminal deletion. Similar deletions of PPR motifs in PRORP1 also inactivated the enzyme [66,111]. Furthermore, the active site of *At*PRORP2 also does not contain metal density and makes interactions with a neighboring molecule (Figure 5B) [106], similar to the active site of MRPP3 [107]. In the case of *At*PRORP2 this is considered an artifact of crystallization, as *At*PRORP2 has activity comparable to *At*PRORP1 *in vitro* [106].

Both groups attempted domain swaps with *At*PRORP1, yet exchanging the NYN domain did not restore activity for MRPP3 alone and was detrimental to the activation by MRPP1/2 [69]. A larger swap, including the central domain and the final helices of the PPR domain, restored some cleavage activity in the absence of MRPP1/2 [107]. The structural and NYN sequence similarities between PRORP1 and MRPP3 probably indicate similar catalytic mechanisms, though more information on MRPP3 structure and catalysis is required to allow a full comparison.

## 4. Catalysis by RNase Ps

RNase P enzymes are metal ion-dependent endonucleases. More specifically, they are hydrolases that catalyze site-specific phosphodiester bond hydrolysis primarily within pre-tRNA. Research into the mechanisms of RNase P catalysis, including the modes of nucleophile activation, have provided insight into the diverse evolutionary backgrounds capable of performing this fundamental biological reaction.

### 4.1. Kinetic Mechanism

**Scheme 1:**
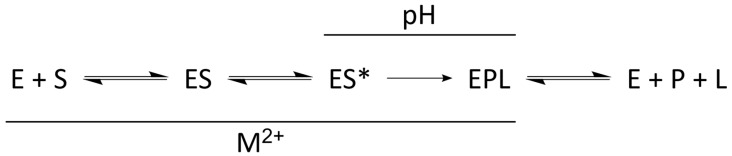
Minimal kinetic mechanism of bacterial RNase P ribozyme catalysis. The initial binding step is bimolecular and dependent on the concentrations of both E and S. Binding, conformational change, and substrate hydrolysis are all dependent on divalent metal ions (M^2+^), while hydrolysis is also dependent on pH.

Transient kinetic studies of *B. subtilis* RNase P established a minimal four-step kinetic mechanism (Scheme 1) [34,112]. In this mechanism, RNase P (E) and pre-tRNA (S) associate in a two-step binding event in which they first form an enzyme–substrate complex (ES) in a near diffusion-limited binding step. Once bound, the ES complex isomerizes to a catalytically-competent conformer (ES*) in a metal-dependent step, referred to as the “conformational change” step. Pre-tRNA is then cleaved to form mature tRNA and 5’ leader and the products dissociate. Product release is rate-limiting for the bacterial enzyme, resulting in a kinetic burst under multiple turnover conditions [113].

At low pH and under single turnover conditions, the observed rate constant (*k*_obs_) increases with a log-linear relationship to pH [112]. The Hill coefficient, *n*_H_ = 1, is consistent with a single ionization producing the metal-hydroxide nucleophile required for catalysis [112]. This is in contrast to nucleases that utilize a general base, which display *n*_H_ = 2 [114,115]. The conformational change step becomes rate-limiting at high pH, resulting in a kinetic (rather than thermodynamic) p*K*_a_ [112]. In the two-step binding mechanism, ES* is stabilized by at least two classes of inner-sphere metal ions [34]. A class of high-affinity divalent cations is required to stabilize the ES* conformer, while a lower-affinity metal ion activates catalysis [32,34,35,116].

The conformational change has numerous potential functions for catalysis. It has been proposed to act as a proofreading step, allowing RNase P to recognize cognate substrates and distinguish them from non-cognate substrates [34,117,118]. This includes facilitating a proposed unwinding of the 5’ and 3’ ends of pre-tRNA [119]. For instance, the nucleotide base at the N(-1) position in the 5’ leader has been proposed to interact with a highly conserved adenosine in RNase P RNA to strengthen the fidelity of pre-tRNA cleavage (see Section 5.1) [120], which would require a structural rearrangement [121,122].

In fact, time-resolved fluorescence resonance energy transfer (trFRET) data showed that during the transition from ES to ES*, the 5’ leader of pre-tRNA moves 4–6 Å closer to the RNA–protein interface. This movement suggests that the substrate is repositioned in the active site during the ES– ES* transition [34]. In the ES* complex, the central cleft of the RnpA protein binds to the 5’ leader of the pre-tRNA substrate between N(-4) and N(-7), extending the leader and decreasing its structural dynamics to position the cleavage site [121,123,124].

The conformational change could allow the C- and S-domains to position functional groups and catalytically important ions in the active site [125], as well as position the active site with respect to substrate for catalysis [126,127]. For instance, in the solution structure of *B. stearothermophilus*, RNase P RNA is in a conformation that would not be suitable to dock with the substrate, suggesting that the conformation of RNase P RNA changes upon binding RnpA, metal ions, and pre-tRNA [84,128,129]. Of note, the crystal structure of the *T. maritima* RNase P may not reflect the active ES* conformation as it is a product complex and does not contain pre-tRNA [22].

### 4.2. RNase P Ribozyme Metal-Binding Sites

Identifying the position of metal ions involved in catalysis in RNase P RNA is challenging because the majority of divalent metal ions bind nonspecifically via electrostatic interactions [130]. Additionally, the P RNA requires divalent ions for stabilizing the folded structure [131,132,133]. Further, crosslinking studies of *E. coli* RNase P RNA examining the position of the pre-tRNA cleavage site relative to the P4 helix suggest that metal ion-binding in the P4 helix indirectly stabilizes catalytic metal ions that interact with the scissile phosphodiester bond [134]. Evidence for the position of catalytic metal ions includes biochemical data from single-atom modifications and functional group substitutions in nucleobases, sugars, and phosphate oxygens [135,136,137,138,139,140,141,142,143].

Important early work from Pace and colleagues identified specific sites on the P4 helix of the bacterial P RNA that are important for catalysis [135,138]. Specifically, rescue of phosphorothioate substitutions with Mn^2+^ or Cd^2+^ revealed that the non-bridging phosphodiester oxygens of P4 helix residues A50 and G51 coordinate to the catalytic metal ions through inner-sphere interaction [142]. These experiments suggested metal coordination through the pro-*S*_P_ oxygen of A50 (in the P RNA but not the holoenzyme), and to both pro-*R*_P_ and pro-*S*_P_ oxygens of G51 [141,142]. Recent biochemical experiments showed that 4-thiouridine substitution at the universally conserved U52 decreases the cleavage rate constant and addition of Cd^2+^ can partially rescue this defect, suggesting that the carbonyl oxygen at the O4 position coordinates a metal ion through an inner-sphere interaction (X.L., Y.C., C.A.F., unpublished data) [7,144]. Each of these metal-coordination sites is consistent with the crystal structure of the *T. maritima* holoenzyme product complex, which includes two metals bound to the active site (Figure 4C) [22].

Using single-atom modifications, additional metal-binding sites have been proposed on the bacterial P RNA. A 7-deaza-2’-deoxyadenosine substitution of either A48 or A49 further decreased the cleavage rate constant [141]. These data suggest that the bases and backbone of A48, A49, and A50 participate in stabilizing a catalytically important metal ion-binding site.

Additional catalytic metal sites were identified on the pre-tRNA substrate. Both *R*- and *S*-phosphorothioate substitutions at the scissile phosphodiester bond of the pre-tRNA substrate decrease the cleavage rate constant catalyzed by RNase P RNA by 10^3^-fold–10^4^-fold. The activity of the *R_­_*-phosphorothioate-substituted pre-tRNA can be partially rescued by addition of thiophilic metal ions (Mn^2+^ and Cd^2+^) [136,137,139,140,143]. Such rescue experiments provided evidence for inner-sphere coordination of two metal ions to the pro-*R*_P_ oxygen of pre-tRNA. At the N(-1) position of pre-tRNA, 2’-NH_2_ and 2’-H substitutions decrease both the catalytic rate constant and the Mg^2+^ affinity for RNase P RNA. Mn^2+^ can rescue cleavage of both 2′-NH_2_ and 2′-H pre-tRNA, suggesting that the 2’-OH of pre-tRNA at the cleavage site also interacts with a metal-bound water molecule [143].

### 4.3. Hydrolysis Requires Activation of a Metal-Bound Water Molecule

**Scheme 2:**
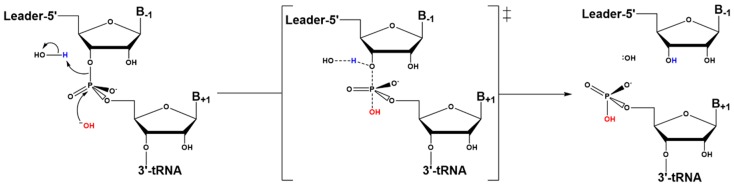
The concerted hydrolytic mechanism proposed for bacterial RNase P. The acid (A) may be a water or metal-bound water.

Nucleophile activation in RNase P catalysis is proposed to proceed by stabilizing a metal-bound hydroxide. Kinetic isotope effect studies on RNase P RNA catalysis conducted by the Harris group suggested that a metal-bound hydroxide, which is not deprotonated by a general base, serves as the nucleophile in the reaction (Scheme 2) [145]. The isotope effect of the reaction of RNase P with pre-tRNA in 50% ^18^O-labeled water was compared to that of two model systems: 1) hydrolysis of thymidine 5’ p-nitrophenyl monophosphate (T5PNP) catalyzed by Mg^2+^, which occurs via a concerted mechanism with a pentacoordinate transition state; and 2) conversion of adenosine to inosine catalyzed by adenosine deaminase (ADA), which uses a stepwise mechanism with formation of a tetrahedral intermediate [145]. The data indicated that the bonding environment in the transition state was more akin to that of T5PNP hydrolysis [145,146].

### 4.4. RNase Ps Utilize Similar Catalytic Strategies

Magnesium ions (Mg^2+^) activate RNase P catalysis *in vitro* and *in vivo* [3,131]. The *T*. *maritima* RNase P product complex crystal structure visualized two potential metal sites [22], yet the number of metals involved in the active site chemistry remains in question. Mechanistic models with three activating metal ions have also been proposed, including a co-catalytic metal bound near the 2’-OH on N(-1) of the substrate The general two-metal ion mechanism proposed for nucleases and polymerases over 20 years ago has largely been vindicated by subsequent studies and is consistent with the PRORP mechanism [110,147,148,149]. The general two-metal ion mechanism includes metal 1 positioning and activating a hydroxide nucleophile, while metal 2 stabilizes the transition state and coordinates a water molecule to protonate the 3’ oxyanion leaving group [22,136,137,147,150]. Additionally, both metal ions are proposed to stabilize the developing charge in the transition state.

PRORP1 from *A. thaliana* is the PRORP best described mechanistically. It has been used as a model system to study mammalian mitochondrial RNase P, given that it is homologous to the nuclease subunit of the human enzyme. The mechanistic data available enables a detailed comparison between the protein-only and the bacterial ribozyme RNase P enzymes. In contrast to the ribozyme, data suggest that the active site metals of PRORP1 do not contact the pro­-*R*_P_ oxygen of the scissile phosphate [151]. Experiments with substrates containing an *R*-phosphorothioate at the scissile phosphate indicated only a five-fold reduction in Mg^2+^-dependent PRORP activity, compared to the >26000-fold decrease for the ribozyme [151]. In PRORP the pro-*S*_P_ oxygen of the scissile bond of pre-tRNA is proposed to contact a metal ion based on the absence of a pro-*R*_P_ effect and by homology to other nucleases [110,151,152].

**Figure 7:**
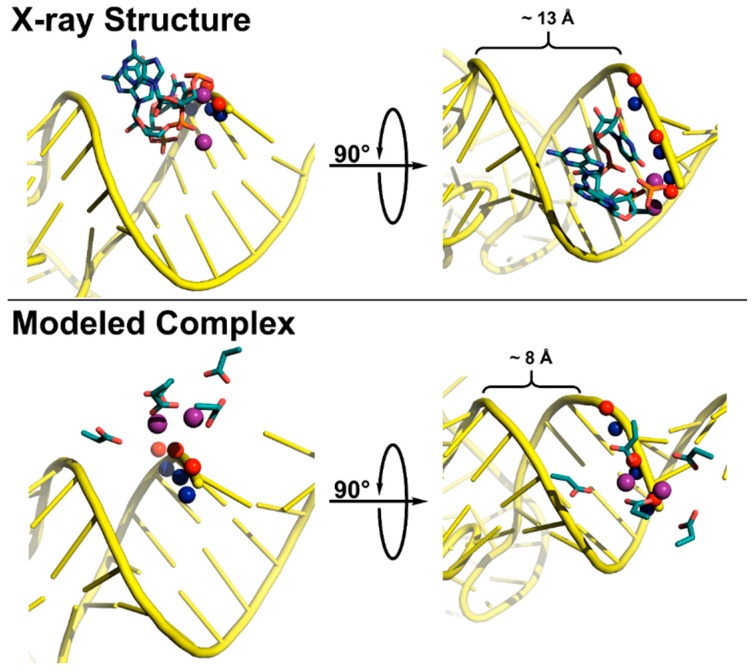
Active site coordination of substrate by *T*. *maritima* RNase P (top, x-ray crystal structure) and *A*. *thaliana* PRORP1 (bottom, complex modeled in PyMOL). The minor groove width was measured as the distance between the non-bridging phosphate oxygens. **Top**: *T*. *maritima* product complex crystal structure (PDB 3q1r) [22]. Pro-*R*_P_ (blue spheres) and pro-*S*_P_ (red spheres) oxygen atoms of tRNA product (yellow cartoon) shown for N(+1)–N(+3). Active site metal atoms (purple spheres) and metal-coordinating residues A50, G51, and U52 are visualized (teal sticks). **Bottom**: The PRORP1 active site (PDB 4g24) was aligned to *S*. *cerevisiae* tRNA^Asp^ (PDB 2tra) using the human DNA exonuclease I active site bound to DNA (PDB 3qeb) as a guide [64,153,154]. The tRNA, backbone oxygen atoms, active site metal atoms, and active site residues D399, D474/475, D493, and D497 are colored as in the top panel.

The difference in metal coordination at the scissile phosphodiester may be explained by altered stereochemical requirements for coordinating the pro-*R*_P_ or pro-*S*_P_ oxygen atoms (Figure 7). In the canonical cloverleaf structure, the pro-*R*_P_ oxygen of the pre-tRNA substrate faces inwards towards the minor groove. Therefore, metal coordination of this oxygen in the ribozyme active site requires insertion of P RNA into the minor groove of the tRNA acceptor stem (Figure 7, top). The result is that the groove is ≥5 Å wider in the ribozyme-bound structure than in a typical tRNA (Figure 7, compare top right to bottom right). The PRORP active site is relatively flat, such that a similar insertion to position the metals near the pro-*R*_P_ oxygen would require large changes in the NYN structure and/or extensive distortions to the pre-tRNA acceptor helix. The small effects of the *R*-phosphorothioate substitution at the scissile bond of pre-tRNA argue against these distortions in a PRORP-substrate complex [151].

Like the ribozyme, a single ionization (*n*_H_ = 1) with increased activity at high pH is observed in the pH-dependence of *A*. *thaliana* PRORP1 [110,112]. Furthermore, no decrease at high pH was observed in the range tested, indicating that no group with a p*K*_a_ ≤ 9.5 acts as a general acid [110]. This might be surprising, given that a frequent component of the RNA world hypothesis posits that protein replaced RNA as the catalytic molecule due to the larger suite of side chains capable of participating in chemistry under biological conditions [10]. Alanine mutations of four conserved aspartates (D399, D474, D475, and D493) significantly reduced activity [66]. Two of the mutants (D474A and D475A) were rescued by increasing the metal concentration [110]. The other two mutants (D399A and D493A) were not rescued, which may indicate they have catalytically-important functions in addition to metal binding [110], though these have not been elucidated. Mutation of D497 has not been attempted, but in the crystal this side chain is positioned to form an outer sphere interaction with metal 2 [110].

**Figure 8:**
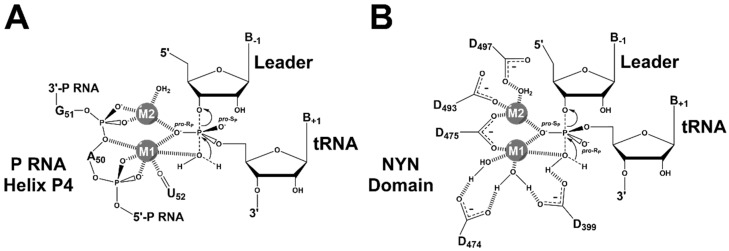
Proposed transition state structure of the catalytic mechanisms of (**A**) bacterial RNase P (as proposed in [22]) and (**B**) *At*PRORP1 (adapted from [110]).

The catalytic efficiencies achieved by PRORP enzymes [71,110,155,156] are comparable or as much as 10^3^ lower than those reported for RNA-based RNase Ps [31,157], inconsistent with an RNA world model in which protein enzymes evolved due only to catalytic enhancements over their RNA world predecessors. In the case of RNase P, it appears that both the RNA-based and protein-only enzymes function to catalyze phosphodiester bond hydrolysis mainly by correctly positioning hydrated metal ions at the cleavage site (Figure 8).

## 5. Substrate Recognition by RNase Ps

**Figure 9:**
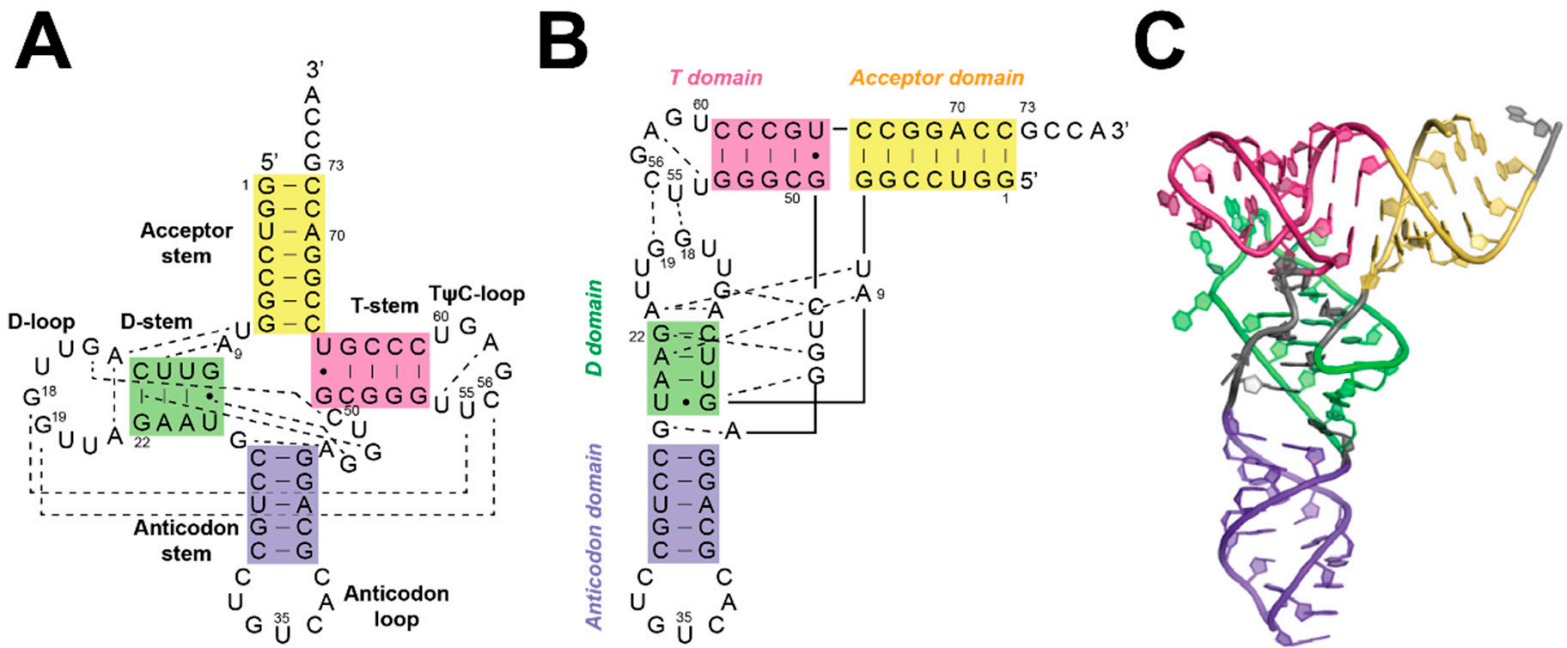
Secondary and tertiary structures of canonical tRNA (adapted with permission from [7]). (**A**) and (**B**) Secondary structure of *B*. *subtilis* tRNA^Asp^ with tertiary interactions denoted by dashed lines. (**C**) A crystal structure of an unmodified *E*. *coli* tRNA^Phe^ (PDB 3l0u) [161].

Most tRNAs from all domains of life have a cloverleaf secondary structure and an L-shaped tertiary structure (Figure 9) [158]. In metazoan mitochondria, many tRNAs deviate from the canonical primary and secondary structure and these tRNAs can be divided into four groups [159]. One type is similar to canonical tRNAs with conserved tertiary interactions, while the other three groups have truncated structural elements or entirely lack certain structural features, resulting in the loss of conserved interactions. These non-canonical tRNA structures led to the proposal that the additional subunits of human mtRNase P evolved to recognize these substrates [160].

### 5.1. Recogntion by Bacterial RNase P

Both the RnpA protein and P RNA subunits contribute to recognition of pre-tRNA substrates. The holoenzyme has a higher binding affinity for pre-tRNA relative to tRNA [31]. The holoenzyme affinity for pre-tRNA varies with the leader length from 2- to 5-nt [116,124]. This trend was not observed for P RNA alone [116,124], which provided evidence that RnpA enhances substrate affinity and cleavage activity of RNase P by interacting with the 5’ leader. Interactions with the 5’ leader also increase the affinities of Mg^2+^ ions bound to the RNase P—pre-tRNA complex and result in uniform binding affinity for various pre-tRNA substrates by combining a low affinity tRNA body with a high affinity leader sequence [32,117]. Substrate competition kinetics revealed a relatively narrow range of catalytic efficiencies, suggesting that the relative rates of pre-tRNA processing by RNase P are uniform in cells [162]. Cross-linking and fluorescence resonance energy transfer (FRET) experiments first demonstrated a direct interaction between the 5’ leader and RnpA [123,124], and the leader position observed in the *T. maritima* RNase P complex structure is consistent with these biochemical data [22].

**Figure 10:**
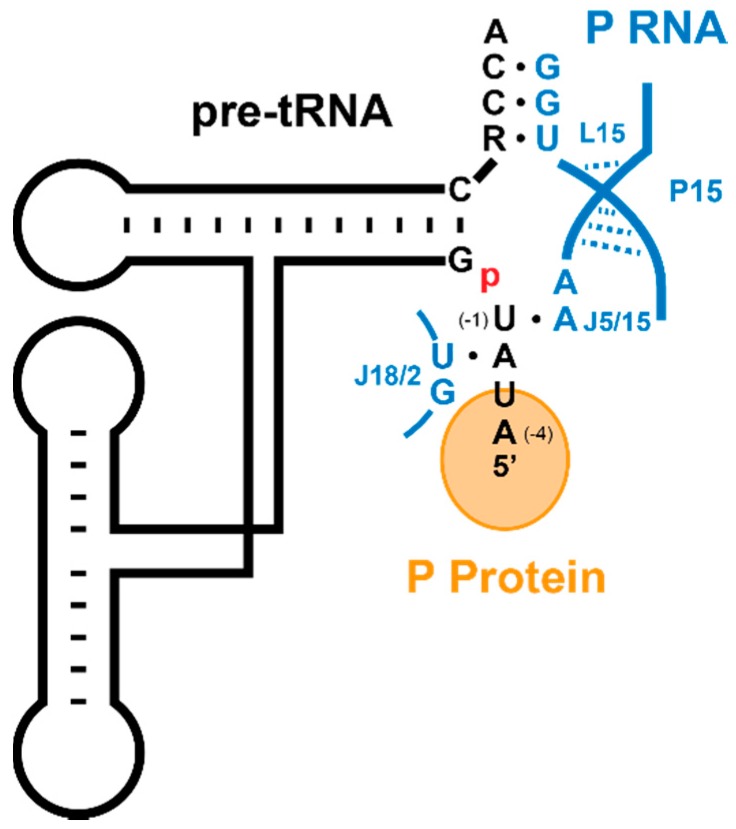
Sequence specific interactions between 5’ and 3’ sequences of pre-tRNA and bacterial RNase P (adapted with permission from [7]). The 3’ RCCA and U(-1) base pair with the GGU motif in the L15 loop and A213, respectively, while A(-2) makes a non-Watson-Crick interaction with U294 [23,120,175].

Sequence-specific interactions between bacterial RNase P and substrates have been identified in both the 5’ leader sequence flanking the tRNA genes and the 3’ RCCA motif (Figure 10) [120,163,164,165,166,167,168]. These interactions enhance pre-tRNA affinity and cleavage. The 3’ RCCA motif of tRNA forms Watson-Crick base pairs with a GGU sequence in the P15 loop (L15), which enhances pre-tRNA and Mg^2+^ affinity in P RNA and to a lesser extent in the holoenzyme [169,170,171,172,173]. The 3’ RCCA interaction motif is missing in archaeal-type M P RNA and eukaryotic P RNA, consistent with the lack of the genomically-encoded 3’ RCCA motif in pre-tRNA genes in these organisms [174].

In both *E. coli* and *B. subtilis* RNase Ps, biochemical studies identified a sequence preference for uracil at position N(-1) in the 5’ leader of pre-tRNA. There is a proposed base pairing between this nucleotide and A213 in J5/15 of P RNA [120,175]. Statistical analyses of the sequences of pre-tRNA genes for these species also reveal a preference of U(-1) [168]. *B. subtilis* RNase P displays a preference for A(-2), forming a trans Watson-Crick–sugar edge interaction with P RNA G319 (U294 in *T*. *maritima*) [23]. Consistent with these observations, there is also a statistically significant preference of A(-2) in *B. subtilis* pre-tRNA genes, suggesting there may be an important interaction to P RNA at this position [23,168].

Bioinformatic analyses of pre-tRNA genes from 161 bacterial species indicate that 91% have statistically significant nucleotide preferences in the 5’ leader region [168]. Nucleotides N(-1) to N(-4) have the highest level of preference, with an increasing preference closer to the RNase P cleavage site. These genomic data also suggest that the sequence preferences vary by bacterial species [168]. These results together suggest that sequence-specific recognition between RNase P and pre-tRNA 5’ leaders are widespread, but diverse among bacterial species. Consistent with this, a newly developed high-throughput sequencing kinetics approach (HITS-KIN) analyzing thousands of variants revealed that *E. coli* RNase P has pronounced sequence preferences in the 5’ leader sequence [176].

The D–TψC interaction is an important factor for pre-tRNA recognition and cleavage site selection by bacterial RNase P. The *E*. *coli* RNase P holoenzyme can tolerate model hairpin-loop substrates that mimic the acceptor and TψC-stems, as well as shorter three base pair hairpin loops, but these are miscleaved at a higher frequency than more complete substrates [127,177]. Deletion or elongation of the D-stem of a cyanobacterial pre-tRNA^Gln^ substrate led to a considerable extent of miscleavage at N(-1) by the *E. coli* RNase P holoenzyme [178]. These results are consistent with structural data indicating interactions between CRII–III and the D–TψC loops [22].

### 5.2. Substrate Recogntion by Archaeal and Eukaryotic RNase P

In archaeal RNase P, the protein subunits and divalent metal ions affect cleavage fidelity and catalytic efficiency [50,178]. For *P*. *furiosus* (*Pfu*) P RNA, miscleavage of model substrates is reduced either by complexation with four proteins (RPP21–RPP29 and POP5–RPP30) or in the presence of high Mg^2+^ concentration [50]. While a long hairpin and mini-hairpin model substrates can be cleaved by *Pfu* P RNA alone, a mini-hairpin substrate with an N(-1)–N73 wobble base pair is not detectably cleaved by *Pfu* P RNA, even upon addition of the RPP21–RPP29 complex [50].

For human nuclear RNase P, a linker sequence of at least one nucleotide between the accepter-stem and TψC-stem in a model substrate was needed for catalytic activity [179]. Substrates lacking either the anticodon stem-loop or most of the variable region are cleaved by human nuclear RNase P, but substrates lacking both of these features are not [179]. These data suggest that either the substrate variants with more extensive truncations were misfolded or this enzyme recognizes different tertiary structure features of tRNA than bacterial RNase P, which tolerates extensive truncations to the tRNA structure [179]. In addition, insertions of extra base pairs (2-bp) in either the accepter-stem or the T-stem in model substrates resulted in miscleavage at +3 or +2 positions by human nuclear RNase P, but did not affect the fidelity of *E. coli* P RNA [179]. These data led to a hypothesis that eukaryotic RNase P likely uses a more strict “measuring mechanism” to recognize the cleavage site [174]. The nuclear yeast RNase P is able to bind and cleave single-stranded RNAs *in vitro* and mutation to the P RNA resulted in accumulation of intron-containing pre-mRNAs and non-coding RNA transcripts, suggesting that RNase P is involved in the turnover of those RNA intermediates [180,181].

### 5.3. Substrate Recogntion by Protein-Only RNase Ps

Most plant tRNAs have canonical secondary and tertiary structures [182,183], so it is not unexpected that *A. thaliana* PRORPs efficiently cleave bacterial substrates such as *Thermus thermophilus* pre-tRNA^Gly^ [151] and *B*. *subtilis* pre-tRNA^Asp^ [110]. *At*PRORP2 also cleaves the *T*. *thermophilus* pre-tRNA^Gly^ bacterial substrate at 28 ˚C, but not at 37 ˚C [151], which suggests that structural dynamics of either pre-tRNA or PRORP2 are important for recognition and catalytic efficiency. Furthermore, substrate recognition strategies of the three *At*PRORP isozymes is similar, as catalytic efficiencies with four pre-tRNA substrates varied <10-fold [156]. Although the binding affinity for pre-tRNA substrates varies by up to 100-fold, all three *At*PRORPs have similar affinities for a given substrate [156]. Additional substrates should be screened before these results are extrapolated, as this uniformity may not be true of all substrates in the much larger pool of plant pre-tRNAs. This could include an experiment such as HITS–KIN, with a mixture of substrates in a large-scale assay [176].

Recent biochemical studies also demonstrate that *At*PRORPs do not make contacts with the 3’ trailer or beyond N(-1) or N(-2) of the leader that significantly enhance affinity or cleavage rates, therefore recognition determinants are located primarily in the tRNA body [155,156]. Deletion of the D- and anticodon-stems or mutation of the conserved G18 nucleotide in the *A. thaliana* pre-tRNA^Cys^ abolishes PRORP1-catalyzed cleavage under multiple-turnover conditions [184]. However, deletion or elongation of the D-stem in the cyanobacterial pre-tRNA^Gln^ substrate did not affect cleavage by partially purified *A. thaliana* RNase P under multiple turnover conditions [178]. These data argue that the D-loop contribution for PRORP recognition may be dependent on the substrate context or stability.

As expected, the PPR domain is important for substrate recognition by *At*PRORP1. Deletion of all or parts of the PPR repeats decreases substrate affinity by up to 30-fold and abolishes catalysis [66,111]. Conserved nucleotides in D- and TψC-loops (G18, G19, C56, and C57) of mitochondrial pre-tRNA^Cys^ were protected by PRORP1 in footprinting experiments, leading to the hypothesis that the PRORP1 PPR domain contacts this region [184]. Other PPR proteins have been implicated in single-stranded RNA (ssRNA) binding with proposed base-specific recognition motifs utilizing residues on two subsequent helices [185,186]. Docking and mutagenesis experiments with PRORP1 suggest that N136 in PPR2 and T180 in PPR3 are involved in substrate recognition [111]. Additional mutagenesis studies implicated *At*PRORP3 R145 (*At*PRORP1 R212) in PPR4 in substrate recognition [155]. However, contacts between these amino acids and the pre-tRNA substrate are not yet clear [155]. Given that many of the D- and TψC-loop bases are typically positioned facing into the structure, making hydrogen bonding and/or stacking interactions, and that PRORPs must recognize all tRNAs, it is likely that PRORP PPRs use a recognition strategy alternative to the ssRNA-binding PPR proteins.

*At*PRORP cleavage site selection is less robust than for bacterial RNase P *in vitro*, resulting in multiple products for various pre-tRNAs [151,156,187]. The data suggest that this reduced cleavage fidelity results from the ability of PRORPs to recognize an acceptor stem extended by an N(-1)–N73 base pair [155,156]. Reduction of the base-pairing potential or removing the discriminator base restores cleavage at the canonical site [155,156]. Furthermore, *At*PRORPs catalyze reprocessing of miscleaved *A*. *thaliana* plastid pre-tRNA^Phe^ to yield the correct mature tRNA [156]. Interestingly, extending the TψC-stem length resulted in a +2 cleavage site, yet reducing the acceptor stem by an equivalent number of base pairs restored cleavage to the correct site [155]. *At*PRORP-catalyzed cleavage of mitochondrial and *E*. *coli* pre-tRNA^His^ occurs mostly at the N(-2)/N(-1) phosphodiester bond, thus primarily producing the correct mature tRNA^His^ [155,187].

Canonical pre-tRNA species are generally cleavable by most RNase P enzymes, with the exception of the nuclease subunit of human mtRNase P (MRPP3) [188]. The additional human mtRNase P subunits (MRPP1 and MRPP2) are essential for efficient catalytic activity of this enzyme and may be particularly important for recognizing non-canonical mt-tRNAs. Knockdown of both MRPP1 and MRPP2 has a large effect on mitochondrial pre-tRNA processing [60,189]. The MRPP1/2 complex catalyzes *N*1 methylation of both G9 and A9, for example, in mt-tRNA^Ile^ and mt-tRNA^Lys^ [68]. However, experimental data suggest that the methyltransferase activity of MRPP1 and the catalytic activity of MRPP2 are not essential for activation of MRPP3 cleavage [68]. The MRPP1 methylation site at R9 is normally buried in the canonical tRNA structure, making reverse-Hoogsteen triple-base interactions with the N12–N23 base pair [190,191,192]. The *N*1 methylation of A9 in (mt)tRNA^Lys^ switches the primary transcript from an extended hairpin to a canonical cloverleaf structure [193,194,195]. Whether recognition of A9 or G9 by MRPP1 facilitates cleavage by MRPP3 by provoking a conformational change in mt-tRNA, has not been determined [196,197].

### 5.4. Non-tRNA RNase P Substrates

In addition to pre-tRNA, RNase Ps catalyze processing of various other substrates in cells (see [118] for review). In Bacteria, RNase P processes pre-4.5S RNA [198,199], pre-transfer-messenger RNA (pre-tmRNA) [200], messenger RNAs (mRNAs) [201,202], riboswitches [203], the lactose operon [204], non-coding OLE RNA [205], and several phage-induced regulatory RNAs [206]. In Eukarya, alternative nuclear RNase P substrates are primarily long non-coding RNAs (lncRNAs), including pre-ribosomal RNA (pre-rRNA) [207], pre-tmRNA [208], hidden-in-reading-frame-antisense 1 (HRA1) [209], and numerous nuclear-encoded yeast RNAs [180]. Additionally, it has been shown in mice and humans that the metastasis-associated lung adenocarcinoma transcript 1 (MALAT1) and multiple endocrine neoplasia β (MENβ) transcripts are processed by nuclear RNase P [210,211]. Few alternative substrates have been identified for PRORPs, which include a tRNA-like structure (t-element) in a precursor *Arabidopsis* mitochondrial transcript and mirror tRNAs in polycistronic transcripts from the antisense strand of metazoan mitochondrial tRNAs [63,189].

In many, but not all, cases of non-tRNA RNase P substrates, the transcripts are proposed to adopt a structure similar to the tRNA elements that are critical for recognition [200,210,211]. For instance, RNase P processes MALAT1 and MENβ at their 3’ ends at tRNA-like structures [210,211]. These structures are processed by both RNase P and RNase Z, generating small cytoplasmic tRNA-like RNAs: MALAT1-associated small cytoplasmic RNA (mascRNA) from MALAT1 and menRNA from MENβ, and a nuclear-retained lncRNA [210]. Recently, RNase P recognition and cleavage sites were found within human liver mRNA transcripts [212]. The results revealed the presence of RNase P recognition sites through competition assays, but found that RNase P did not catalyze cleavage at most of these sites *in vitro* [212]. Evidently, RNase Ps from a variety of evolutionary lineages have developed cellular functions that extend beyond the canonical 5’ end maturation of pre-tRNAs.

## 6. Conclusions

RNase P enzymes represent an extreme case of convergent evolution. While RNA-based RNase Ps and eukaryotic PRORPs have distinct evolutionary origins, there are many similarities in their catalytic and substrate recognition strategies. As was detailed by Lechner and colleagues [11], the emergence of PRORP nucleases in early Eukarya appears to have caused a flurry of diversification. The resulting competition for cellular RNase P function produced remarkable diversity, from a lack of PRORP sequences in Fungi [11], to the loss of the RNA-based RNase P in plants such as *Arabidopsis* [71]. There remain gaps in our understanding of this class of enzymes, including RNase P *in vivo* substrate selectivity and distribution among several eukaryotic clades.

Some aspects of cleavage activity and substrate recognition mechanisms vary between different classes of RNase P enzymes. Dissecting the mechanism of substrate recognition by RNase Ps will be important for designing inhibitors that specifically target RNase P in pathogens while leaving human RNase P activity intact. Further, mechanistic studies of highly purified eukaryotic RNase P enzymes are still needed for a deeper understanding of the role of RNase P in processing pre-tRNA *in vitro* and *in vivo*, including the molecular details of substrate recognition by the protein-only human mtRNase P.

Recent findings have revealed non-canonical cellular functions for eukaryotic RNase P [213]. This includes acting as a transcription factor for RNA polymerases I and III [48,214,215,216]. The chromatin of transcriptionally active tRNA, rRNA, and 5S rRNA genes in human cells directly interacts with nuclear RNase P subunits in a cell cycle dependent manner [215]. Further, the RNP of nuclear RNase P is an essential part of initiation complexes recruited to the 5S rRNA genes in HeLa cells [216]. Additionally, the RPP29 subunit of human nuclear RNase P represses H3.3 deposition in chromatin [217].

In the more than four decades since their initial discovery, much has been learned about the macromolecular composition, catalysis, and substrate recognition of the RNA-based RNase Ps. Likewise, in the nearly eight years since the human mitochondrial PRORP *in vitro* reconstitution was published, much has been learned about the *in vivo* function and domain architecture of the nuclease subunit and its homologs. However, there are still many aspects of catalysis, substrate recognition, and cellular functions that remain to be clarified. We are optimistic that current and future RNase P research will illuminate the evolution and activities of these uniquely diverse enzymes.

## Figures and Tables

**Table 1:** Conservation of RNase P protein components across the domains of life. RnpA proteins from various Bacteria share homology to each other, but not to archaeal or eukaryotic proteins. Archaeal and eukaryotic nuclear RNase P proteins share sequence homology with proteins on the same row. Protein-only RNase P components do not share homology with any of the RNA-based RNase P protein components.

Bacteria	Archaea ^1^	Eukarya			
		RNA-based		PRORP	
		Yeast	Human	Human	Plant
RnpA					
	Mth687p	POP5	hPOP5		
	Mth11p	POP4	RPP29		
	Mth688p	RPP1	RPP30		
	Mth1618p	RPR2	RPP21		
	L7Ae ^2^	POP3	RPP38		
		POP1	hPOP1		
		POP6	RPP25		
		POP7	RPP20		
		POP8			
			RPP14		
			RPP40		
				MRPP1	
				MRPP2	
				MRPP3	PRORP1–3

^1^ Naming for RNase P proteins from *Methanobacter thermoautotrophicus* [59]. ^2^ L7Ae homologs are only associated with M-type archaeal RNase P [47].

## Abbreviations

The following abbreviations are used throughout this manuscript:

RNase P	Ribonuclease P
RNA	ribonucleic acid
tRNA	transfer RNA
pre-tRNA	precursor tRNA
P RNA	RNase P RNA
PRORP	protein-only (alternatively: proteinaceous) Ribonuclease P
MRPP	mitochondrial RNase P protein
NYN	Nedd4-BP1, YacP nuclease
PPR	pentatricopeptide repeat
